# Linsitinib inhibits proliferation and induces apoptosis of both IGF-1R and TSH-R expressing cells

**DOI:** 10.3389/fimmu.2024.1488220

**Published:** 2024-12-11

**Authors:** Maximilian Luffy, Anna-Lena Ganz, Stefanie Wagner, Jan Wolf, Julian Ropertz, Ryan Zeidan, Jeffrey D. Kent, Raymond S. Douglas, George J. Kahaly

**Affiliations:** ^1^ Molecular Thyroid Research Laboratory, Department of Medicine I, Johannes Gutenberg-University (JGU) Medical Center, Mainz, Germany; ^2^ Sling Therapeutics, Ann Arbor, MI, United States

**Keywords:** Linsitinib, small molecule kinase inhibitor, insulin-like growth factor 1 receptor, thyrotropin receptor, thyroid eye disease, apoptosis, cell proliferation, cell-based bioassays

## Abstract

**Background:**

The insulin-like growth factor 1 receptor (IGF-1R) and the thyrotropin receptor (TSH-R) are expressed on orbital cells and thyrocytes. These receptors are targeted in autoimmune-induced thyroid eye disease (TED). Effective therapeutic treatment of TED inhibits activation of the IGF-1R/TSH-R complex.

**Methods:**

The inhibitory effect on cell proliferation of a small molecule targeting IGF-1R phosphorylation (Linsitinib) was investigated in an IGF-1R expressing cell line and a Chinese Hamster Ovary (CHO) cell line overexpressing TSH-R. An IGF-1R monoclonal antibody antagonist, Teprotumumab served as control. Both cell lines were plated in a 96-well format and treated with both compounds for 24 hours. After addition of tetrazolium, absorbance was measured. The apoptosis marker caspase-3/7 activity was measured. The half-maximal inhibitory concentration (IC_50_) of TSH-R-Ab induced stimulation (stimulatory monoclonal antibody, mAb, M22) of the TSH-R cell line was evaluated with a cell-based bioassay for blocking TSH-R-Ab. Cells were treated with ten rising concentrations of either Linsitinib, Linsitinib + Metformin, Teprotumumab, or a blocking TSH-R mAb (K1-70).

**Results:**

Linsitinib strongly inhibited the proliferation of both cell lines at several concentrations: 31,612.5 ng/mL (IGF-1R cell line -78%, P=0.0031, TSH-R cell line -75%, P=0.0059), and at 63,225 ng/mL (IGF-1R cell line -73%, P=0.0073, TSH-R cell line -73%, P=0.0108). Linsitinib induced apoptosis of both cell lines, both morphologically confirmed and with an increased caspase-3/7 activity at concentrations of 31,612.5 ng/mL (IGF-1R cell line P=0.0158, TSH-R cell line P=0.0048) and 63,225 ng/mL (IGF-1R cell line P=0.0005, TSH-R cell line P=0.0020). Linsitinib markedly inhibited proliferation of the IGF-1R cell line at all concentrations compared to Teprotumumab (P=0.0286). Teprotumumab inhibition was significant only at 15,806.25 ng/mL with the TSH-R cell line (-15%, P=0.0396). In addition, in the TSH-R-Ab blocking bioassay, Linsitinib and the tested compounds demonstrated strong inhibition across all ten dilutions (100%).

**Conclusions:**

Linsitinib effectively induces apoptosis and inhibits proliferation of both IGF-1R and TSH-R expressing target cells, therefore demonstrating its therapeutic potential to block the reported crosstalk of the two mediators in autoimmune TED.

## 1 Introduction

The Graves’ disease (GD) specific autoantigen thyrotropin receptor (TSH-R) and the insulin-like growth factor 1 receptor (IGF-1R) are molecular targets on both thyrocytes and orbital fibroblasts (1, 2) playing pivotal roles in the pathogenesis of GD and thyroid eye disease (TED) (3, 4). Both the TSH-R and IGF-1R form a physical and functional signaling complex in orbital fibroblasts upon binding of TSH-R autoantibodies to the TSH-R, facilitating receptor crosstalk (5–7). Signaling through this protein complex leads to subsequent expansion of orbital fat and muscle (8), illustrating its role in TED (9, 10). Importantly, pathogenic signaling at either receptor relies on IGF-1R activity, supporting receptor crosstalk and potential TSH-R transactivation of IGF-1R (7). Several therapeutic compounds were investigated to block crosstalk in TED, with IGF-1R being a key target for managing the disease (11, 12).

The IGF-1R functions as an integral membrane receptor and belongs to the tyrosine kinase family (12, 13). Structurally, IGF-1R is a heterotetramer consisting of two α and β subunits linked by disulfide bonds (12). The extracellular ligand-binding domains are located in the α subunits, while the β subunits contain the tyrosine kinase domain, which is crucial for signaling (7). Binding of ligands IGF-1 and IGF-2 to the receptor stimulates intrinsic tyrosine kinase activity, leading to auto phosphorylation and activation of signaling cascades that enhance cell growth, proliferation, and apoptosis modulation through protein kinase B (AKT) and extracellular-signal regulated kinase (ERK) pathways (14–17).

Linsitinib, also known as OSI-906, is a selective small molecule inhibitor of IGF-1R and to a lesser extent insulin receptor (IR) (18). Its selectivity derives from its unique binding mode to both receptors (18), showing over a 100-fold selectivity compared to more than 100 other kinases (19). Linsitinib demonstrates potent activity and favorable pharmacokinetic properties, with bioavailability exceeding 60% in various pre-clinical species, including mouse, rat, dog, and monkey (16). This small molecule, administered orally, has been tested within a phase 2b randomized, double-masked study in patients with clinically active and severe TED (NCT05276063).

In the present pre-clinical study, for the first time the inhibitory effects of Linsitinib on cell proliferation and apoptosis were investigated in both IGF-1R and TSH-R expressing cell lines. Teprotumumab, a fully human monoclonal antibody (mAb) targeting the IGF-1R, served as control. In addition, the half-maximal inhibitory concentration (IC_50_) of TSH-R-Ab induced stimulation in the TSH-R cell line was evaluated using a cell-based bioassay for blocking TSH-R-Ab, comparing the IC_50_ of Linsitinib to Linsitinib + Metformin, Teprotumumab, and a blocking TSH-R mAb (K1-70). Combined treatment Linsitinib + Metformin was included to evaluate potential synergistic effects, as Metformin modulates key signaling pathways targeted by Linsitinib.

## 2 Materials and methods

### 2.1 Cell proliferation assay

#### 2.1.1 Cell lines and cell culture

The human IGF-1R expressing tumor cell line NCI-H295R (ATCC, Manassas, VA, USA), isolated from the adrenal gland of a patient with adrenal cancer, grows primarily adherent as a monolayer and in suspension. This cell line was continuously cultured in 75 cm^2^ flasks containing 25 mL DMEM: F12 base medium from ATCC under aseptic conditions. This growth medium was supplemented with 0.00625 mg/mL insulin, 0.00625 mg/mL transferrin, 6.25 ng/mL selenium, 1.25 mg/mL bovine serum albumin and 0.00535 mg/mL linoleic acid in the form of ITS+ Premix from Corning (Corning, NY, USA), along with a final concentration of 2.5% Nu-Serum (Corning) containing newborn calf serum, epidermal growth factor, endothelial cell growth supplement, insulin and transferrin. The medium was changed three times per week to maintain high cell quality. The medium from the culture flask was dispensed into two 15 mL falcon tubes and 25 mL of fresh DMEM: F12 medium was added to the culture flask. The falcon tubes were centrifuged at 700 rpm for 5 minutes to obtain a cell pellet, allowing the retrieval of cells in suspension. After resuspension of the cell pellet in 1 mL of fresh DMEM: F12 medium, the cells were transferred back into the culture flask. Cultivation was continued in an incubator at 37°C with 5% CO_2_.

The Chinese Hamster Ovary (CHO) cell line overexpressing the TSH-R (QuidelOrtho, San Diego, CA, USA), was thawed immediately before seeding for the proliferation assay and subsequently incubated in the DMEM: F12 medium under the same conditions as the IGF-1R expressing cell line.

#### 2.1.2 Assay protocol

The Cell Proliferation Assay (Promega, Madison, WI, USA) is a colorimetric method using a tetrazolium compound (MTS) for determining the number of viable cells. The assay procedure (**Supplementary Figure 1**) extends over three days and was conducted under aseptic conditions. Upon reaching a minimum of 80% confluence, the IGF-1R expressing cell line was detached from the culture flask using 3 mL of Trypsin-EDTA (PAN Biotech, Aidenbach, Germany) solution for 10 minutes. The resulting cell suspension, supplemented with 8 mL of complete growth medium, was transferred to a falcon tube and spun at 700 rpm for 5 minutes to obtain a cell pellet. Following resuspension of the cell pellet in 1 mL of fresh DMEM: F12 medium, cell counting was performed using Acridine Orange (Logos Biosystems, Anyang-si, South Korea) to prepare a cell suspension containing 200,000 cells. Subsequently, 100 μL of the cell suspension containing the IGF-1R cell line was pipetted into each of the 20 inner wells in quadruplicate of a 96-well test plate (10,000 cells/well). Similarly, the TSH-R cell line was thawed using a 37°C water bath, mixed with 5 mL of DMEM: F12 medium and 100 μL of the resulting cell suspension was pipetted into each of the 20 inner wells in quadruplicate of the test plate (10,000 cells/well) alongside the IGF-1R cell line (**Supplementary Figure 2**). For the blank control, 100 μL of DMEM: F12 medium was pipetted in quadruplicate into the wells. The test plate was cultivated for 24 hours in an incubator at 37°C with 5% CO_2_.

On the second day, a dilution series with four (n=4) rising concentrations (7,903.125 ng/mL, 15,806.25 ng/mL, 31,612.5 ng/mL and 63,225 ng/mL) of Linsitinib (Sling Therapeutics, Ann Arbor, MI, USA) or Teprotumumab (BioVision, Milpitas, CA, USA), as well as a 1:11 dilution in complete DMEM: F12 medium, was prepared for cell treatment. These concentrations were selected based on pre-experimental data, which demonstrated the most significant inhibition. Subsequently, the contents of the test plate were discarded and 100 μL of fresh DMEM: F12 medium was transferred to each well. Afterward, 100 μL of each Linsitinib or Teprotumumab sample were transferred into the wells according to the pipetting scheme (**Supplementary Figure 2**). The incubation of the test plate was continued for an additional 24 hours in an incubator at 37°C with 5% CO_2_.

Morphological changes were microscopically examined in each well on the third day at a magnification of 100x. Subsequently, a tetrazolium compound from Promega was thawed in a 37°C water bath for 10 minutes. Afterward, 40 μL of the tetrazolium reagent was added to each well of the test plate containing the samples in 200 μL of medium. The test plate was incubated for 4 hours in an incubator at 37°C with 5% CO_2_, after which the absorbance of each sample was recorded at 490 nm (GloMax luminometer, Promega).

### 2.2 Caspase-Glo^®^ 3/7 Assay (apoptosis)

#### 2.2.1 Assay protocol

The Caspase-Glo^®^ 3/7 Assay (Madison, WI, USA) measures activities of caspase-3 and -7 as apoptosis marker. The assay was conducted under the same conditions and followed the same general procedure as the proliferation assay. The main differences are outlined below.

Day 2: After discarding the contents of the test plate, 50 μL (instead of 100 μL) of each Linsitinib or Teprotumumab sample was transferred to 50 μL (instead of 100 μL) of fresh DMEM: F12 medium in the wells according to the pipetting scheme (**Supplementary Figure 2**).

Day 3: Morphological changes were microscopically examined in each well at a magnification of 100x. Subsequently, the caspase buffer was thawed in a 37°C water bath for 10 minutes and the caspase substrate was stored until use at room temperature. The thawed buffer was transferred to the bottle containing substrate and mixed. Afterward, 100 μL of the mixed caspase reagent was added to each well of the test plate containing the samples in 100 μL of medium. Following this, the contents of the wells were mixed using a plate shaker at 300 rpm for 30 seconds. The test plate was incubated for 30 minutes at room temperature protected from light, after which the luminescence of each sample was recorded (GloMax luminometer, Promega).

### 2.3 TSH-R-Ab bioassays

#### 2.3.1 Testing of M22

The 80% effective concentration (EC_80_) of the stimulatory mAb M22 (RSR, Cardiff, UK) was determined using a FDA-cleared cell-based stimulating TSH-R-Ab (TSI) bioassay (Thyretain, QuidelOrtho). The bioassay was performed as previously described (20, 21).

In detail, on the first day, the TSH-R cell line was thawed using a 37°C water bath and mixed with 5 mL of growth medium. Subsequently, 100 μL of the cell suspension was pipetted into each of the 48 inner wells of a 96-well plate (10,000 cells/well) pre-coated with Cell Attachment Solution under laminar flow conditions. The cells were grown for 15 to 18 hours to a confluent monolayer in an incubator at 37°C with 5% CO_2_.

On the second day, a dilution series of M22, testing ten (n=10) rising concentrations (0.1420 – 18.1818 ng/mL), as well as a 1:11 dilution in Reaction Buffer (RB), was prepared in test tubes for subsequent cell treatment. In addition, TSI positive, reference and normal controls were diluted 1:11 in RB. For the 1:11 dilutions, 40 μL of the sample/control was mixed with 400 μL of RB. Subsequently, 100 μL of each M22 and control sample was pipetted in duplicate into the test plate according to the pipetting scheme (**Supplementary Figure 3**). The test plate was then incubated for 3 hours at 37°C with 5% CO_2_. After incubation, the contents of the wells were discarded and 75 μL of luciferase solution was added to each well. The test plate was incubated protected from light for 10 minutes, following which luciferase levels in each well were measured using a luminometer (Tecan, Männedorf, Zürich, Switzerland). The recorded relative light units (RLU) were converted to percentage of specimen-to-reference ratio (SRR %) with a cut-off of ≥ 140 SRR percentage (22, 23).

#### 2.3.2 Compound testing

Next, the IC_50_ of TSH-R-Ab induced stimulation by M22 of the TSH-R cell line was evaluated with a FDA-cleared cell-based blocking TSH-R-Ab (TBI) bioassay (Thyretain, QuidelOrtho). IC_50_ values for Linsitinib, Linsitinib + Metformin, Teprotumumab and a blocking TSH-R mAb (K1-70, RSR, Cardiff, Wales, UK) were determined using this blocking bioassay (24–26).

On the first day, the TSH-R cell line was thawed using a 37°C water bath and mixed with 5 mL of growth medium. Afterward, 100 μL of the cell suspension containing 10,000 cells per well was pipetted into each of the 48 inner wells of a 96-well plate pre-coated with Cell Attachment Solution under laminar flow conditions. The cells were incubated at 37°C with 5% CO_2_ for 15 to 18 hours until confluent growth.

On the second day, a dilution series encompassing ten (n=10) rising concentrations of each compound, along with a 1:11 dilution in RB, was prepared for cell treatment. In addition, TBI positive, reference and negative controls were diluted 1:11 in RB and included in each test plate. For the 1:11 dilutions, 30 μL of the sample/control were mixed with 300 μL of RB. Subsequently, the TSH-R cells were initially treated with 100 μL of each compound and control sample in duplicate (**Supplementary Figure 4**) for 1 hour, followed by 2 hours of stimulation with the previously determined double EC_80_ concentration of M22. Afterward, the contents of the wells were discarded and 75 μL of luciferase solution were transferred to each well. The test plate was incubated protected from light for 10 minutes, after which luciferase levels in each well were recorded using the Tecan luminometer. The measured RLU were reported as percent inhibition of luciferase expression relative to induction with bovine TSH alone, with a cut-off of ≥ 34%.

### 2.4 Statistical analysis

Statistical analysis was carried out using GraphPad Prism software version 9.5.1 (GraphPad Software, Boston, Massachusetts, USA) and/or the GraphPad QuickCalcs Web site: https://www.graphpad.com/quickcalcs/Ecanything1.cfm. P-values for statistical comparisons of each concentration relative to the control in the Cell Proliferation Assay as well as in the Caspase-Glo^®^ 3/7 Assay were calculated using Kruskal-Wallis test followed by Dunnett’s multiple comparisons test. The data are expressed as the fold change relative to the control.

P-values for statistical comparisons of Linsitinib versus Teprotumumab in the Cell Proliferation Assay of the IGF-1R expressing cell line were calculated using the two-tailed Mann-Whitney test. The statistical significance was defined as p < 0.05. Data of the TSH-R-Ab assays were visualized in dose-response curves to determine EC/IC.

## 3 Results

### 3.1 Cell proliferation assay

**Figure 1:**
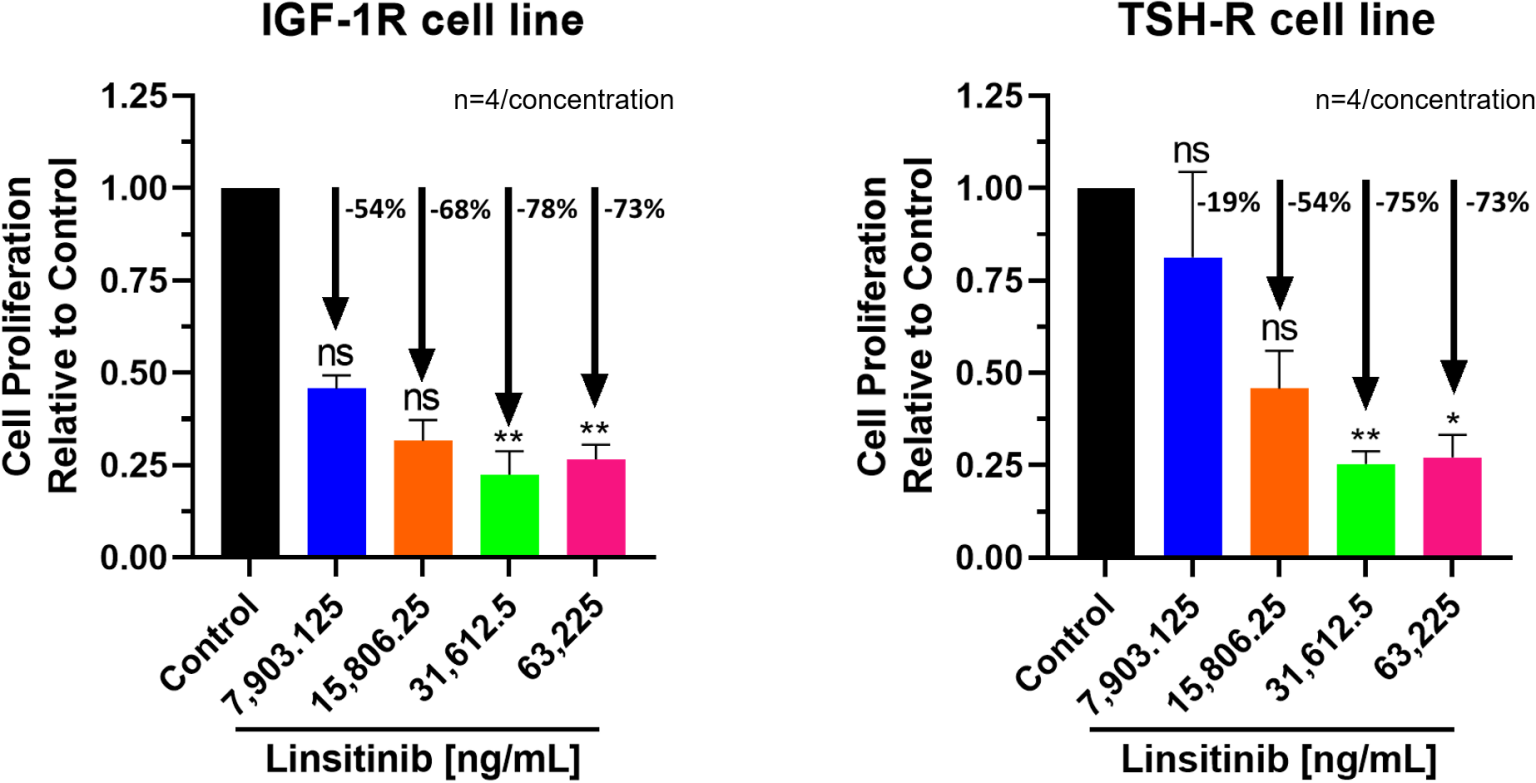
Cell Proliferation Assay of Linsitinib in both IGF-1R and TSH-R cell lines. In the IGF-1R cell line, inhibition ranged from 54 to 78% compared to control, while in the TSH-R cell line, inhibition ranged from 19 to 75%. Significant inhibition was observed at concentrations of 31,612.5 ng/mL (IGF-1R cell line -78%, P=0.0031, TSH-R cell line -75%, P=0.0059), and 63,225 ng/mL (IGF-1R cells -73%, P=0.0073, TSH-R cells -73%, P=0.0108). At lower concentrations (7,903.125 ng/mL or 15,806.25 ng/mL), milder inhibition was noted in both cell lines. Each concentration of Linsitinib was tested in quadruplicate (n=4). ns means P > 0.05. * means P ≤ 0.05, ** means P ≤ 0.01.

Similar, highly significant and marked inhibitory effects of Linsitinib were noted for both IGF-1R and TSH-R expressing cell lines. In the IGF-1R cell line, inhibition ranged from 54 to 78% compared to control, while in the TSH-R cell line, inhibition ranged from 19 to 75% (**Figure 1**). Specifically, substantial inhibition of proliferation was observed at the following Linsitinib concentrations: 31,612.5 ng/mL (IGF-1R cell line -78%, P=0.0031, TSH-R cell line -75%, P=0.0059), and 63,225 ng/mL (IGF-1R cells -73%, P=0.0073, TSH-R cells -73%, P=0.0108). At lower concentrations (7,903.125 ng/mL or 15,806.25 ng/mL), milder inhibition was noted in both cell lines.

**Figure 2:**
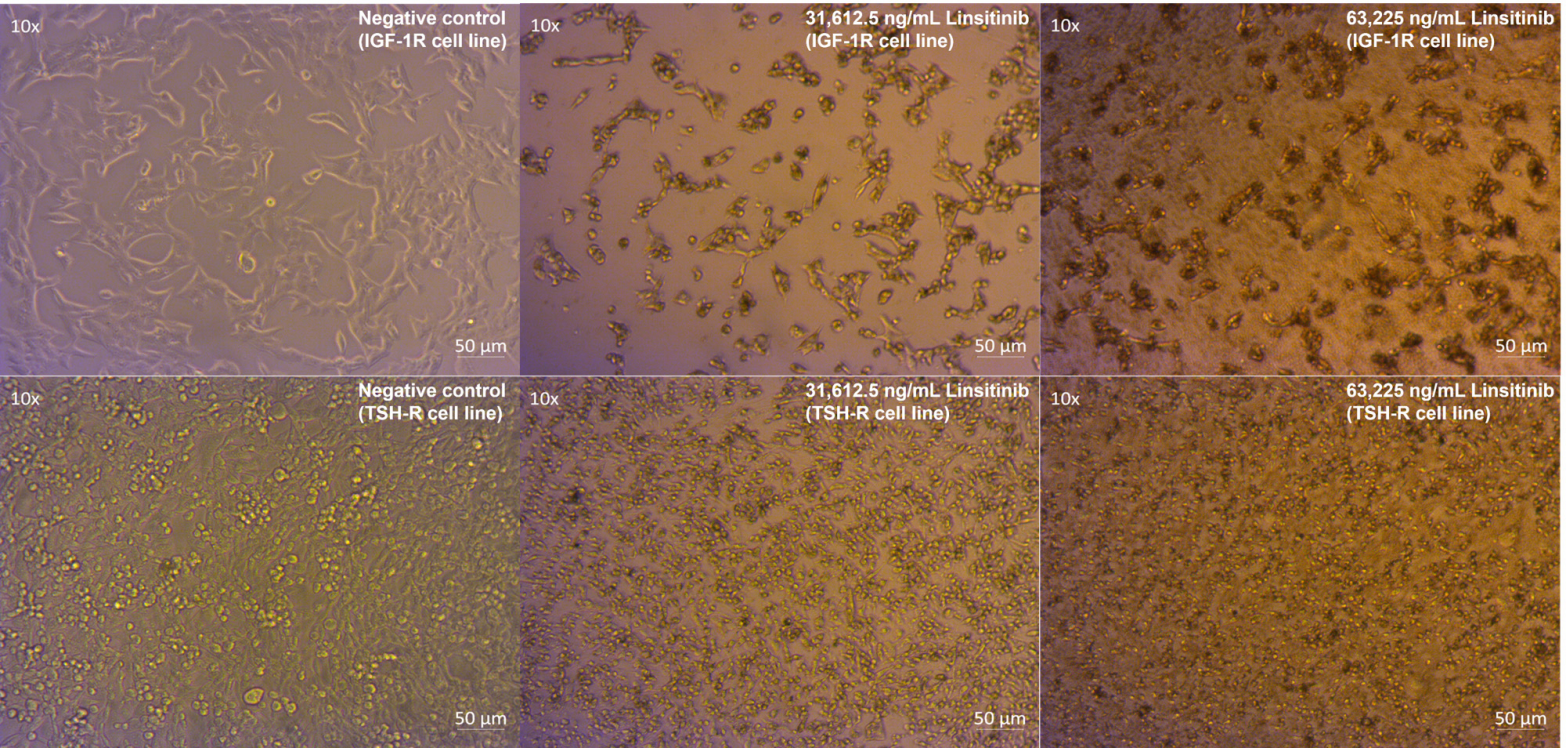
Linsitinib markedly decreases proliferation and induces apoptosis in both IGF-1R and TSH-R cell lines.

Linsitinib induced a microscopically and morphologically confirmed decrease in proliferation across all concentrations, occurring at low (7,903.125 ng/mL) and moderate (15,806.25 ng/mL) concentrations over 24-hour treatment of both cell lines (Zeiss Microscopy, Göttingen, Germany). This inhibitory effect was increased at higher concentrations (31,612.5 ng/mL and 63,225 ng/mL) in both cell lines compared to control (**Figure 2**).

**Figure 3:**
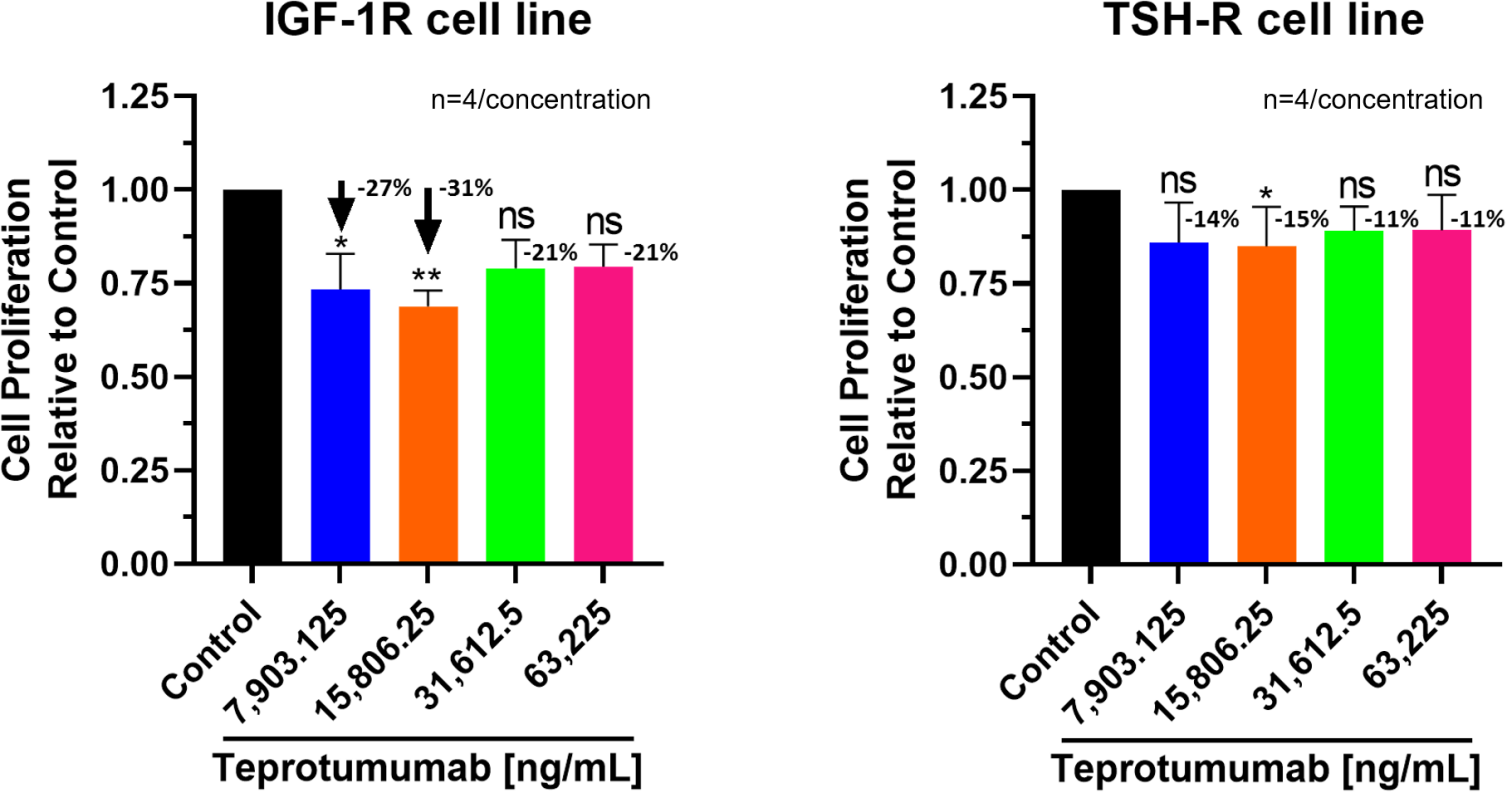
Cell Proliferation Assay of Teprotumumab in both IGF-1R and TSH-R cell lines. In the IGF-1R cell line, inhibition ranged from 21 to 31% compared to control, while in the TSH-R cell line, inhibition ranged from 11 to 15%. Teprotumumab demonstrated significant inhibition of proliferation at 7,903.125 ng/mL (-27%, P=0.0278) and 15,806.25 ng/mL (-31%, P=0.0059) of the IGF-1R cell line, while it was significant only at 15,806.25 ng/mL with the TSH-R cell line (-15%, P=0.0396). Each concentration of Teprotumumab was tested in quadruplicate (n=4). ns means P > 0.05. * means P ≤ 0.05, ** means P ≤ 0.01.

**Figure 4:**
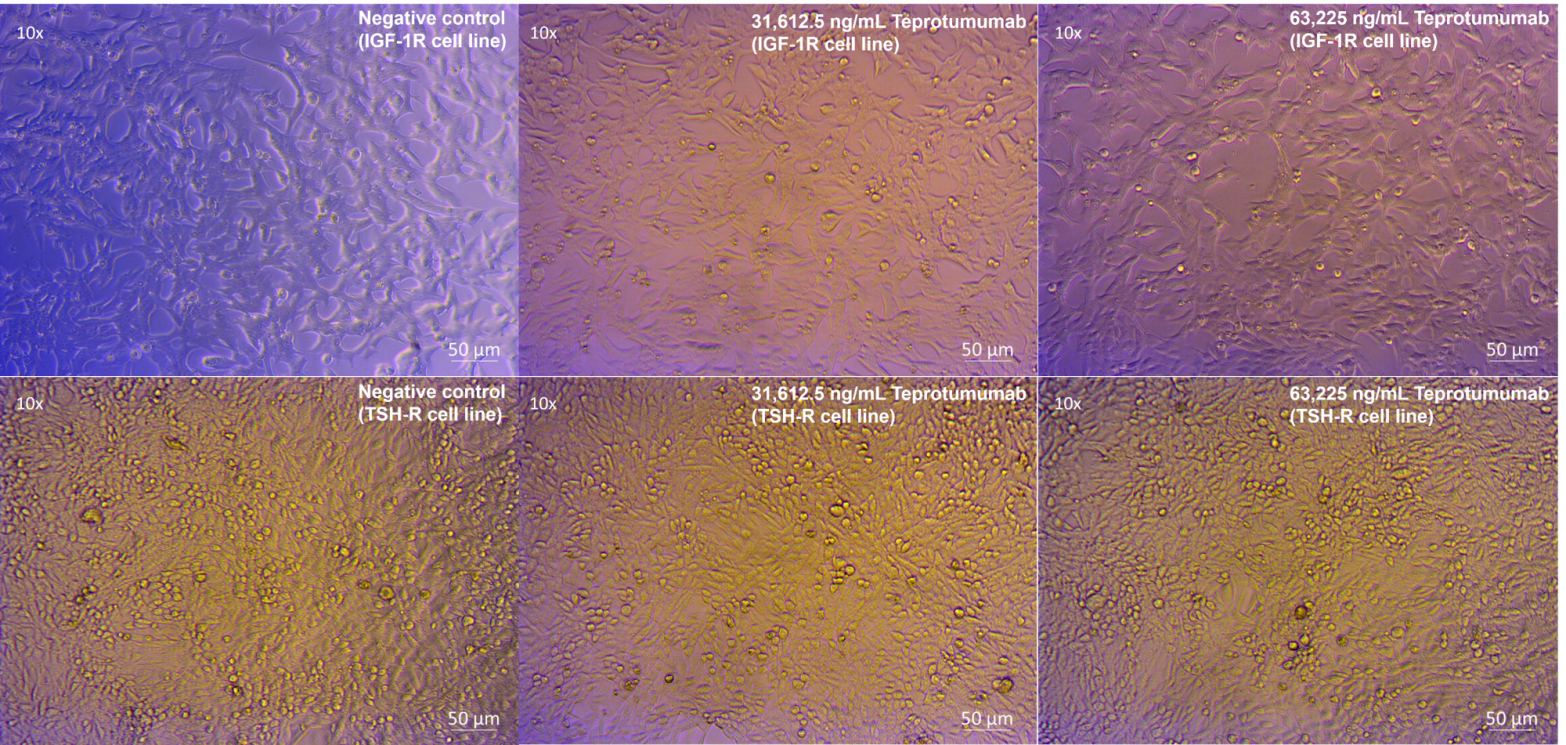
No morphological changes in IGF-1R and TSH-R cell lines following incubation with Teprotumumab, compared to control.

**Figure 5:**
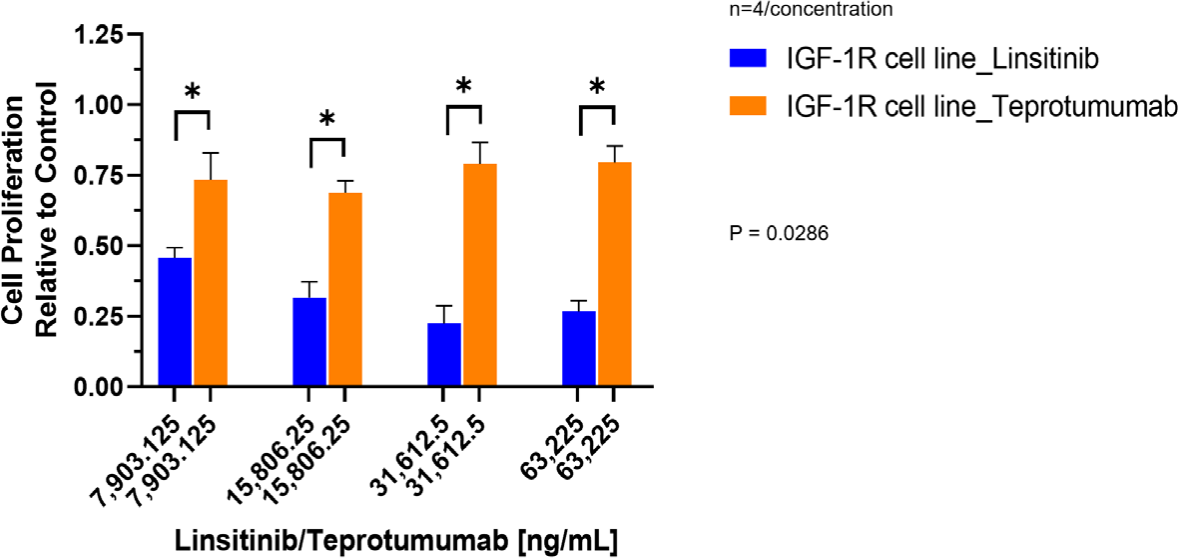
Comparison of Linsitinib and Teprotumumab effects on Cell Proliferation in the IGF-1R cell line. Compared to Teprotumumab, Linsitinib inhibited the proliferation of the IGF-1R cell line markedly more at all concentrations (Linsitinib versus Teprotumumab, P=0.0286). Each concentration of Linsitinib and Teprotumumab was tested in quadruplicate (n=4). * means P ≤ 0.05.

Compared to control, inhibition of proliferation by Teprotumumab ranged from 21 to 31% in the IGF-1R and from 11 to 15% in the TSH-R cell line (**Figure 3**). Teprotumumab demonstrated significant inhibition of proliferation at 7,903.125 ng/mL (-27%, P=0.0278) and 15,806.25 ng/mL (-31%, P=0.0059) of the IGF-1R cell line, while it was significant only at 15,806.25 ng/mL with the TSH-R cell line (-15%, P=0.0396). No morphological changes in proliferation were observed with Teprotumumab across all tested concentrations in both cell lines (**Figure 4**). Compared to Teprotumumab, Linsitinib inhibited the proliferation of the IGF-1R cell line markedly more at all concentrations (Linsitinib versus Teprotumumab, P=0.0286, **Figure 5**).

### 3.2 Caspase-Glo^®^ 3/7 Assay (apoptosis)

**Figure 6:**
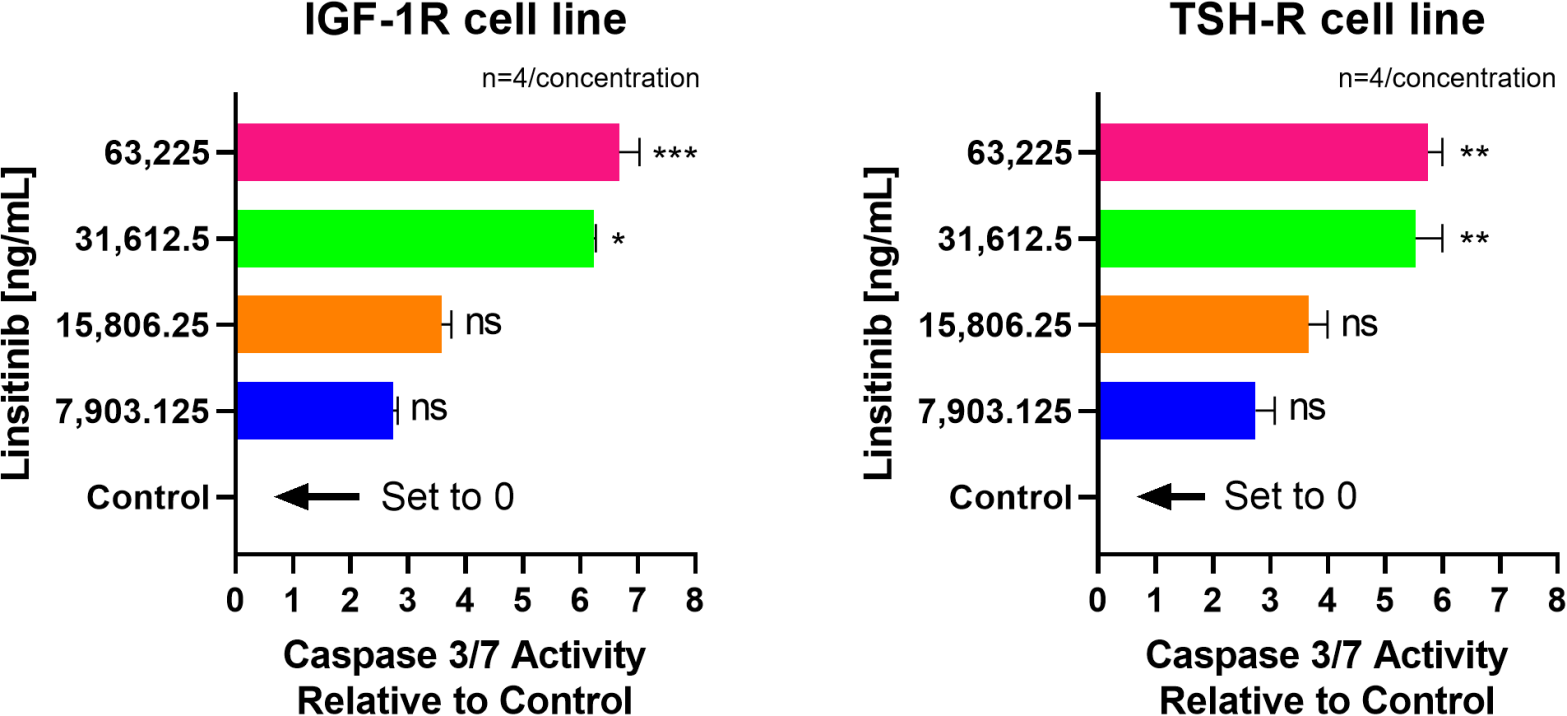
Effect of Linsitinib on Caspase-3/7 Activity in IGF-1R and TSH-R cell lines. In the IGF-1R cell line, caspase-3/7 activity ranged from +2.75 to +6.68 relative to control, while in the TSH-R cell line, caspase-3/7 activity ranged from +2.75 to +5.75 relative to control. Specifically, Linsitinib markedly increased caspase-3/7 activity at concentrations of 31,612.5 ng/mL (IGF-1R cell line +6.24 relative to control, P=0.0158, TSH-R cell line +5.53 relative to control, P=0.0048) and 63,225 ng/mL (IGF-1R cell line +6.68 relative to control, P=0.0005, TSH-R cell line +5.75 relative to control, P=0.0020). At lower concentrations (7,903.125 ng/mL and 15,806.25 ng/mL), no significant increase in caspase-3/7 activity was observed in either cell line. Each concentration of Linsitinib was tested in quadruplicate (n=4). ns means P > 0.05. * means P ≤ 0.05, ** means P ≤ 0.01, *** means P ≤ 0.001.

**Figure 7:**
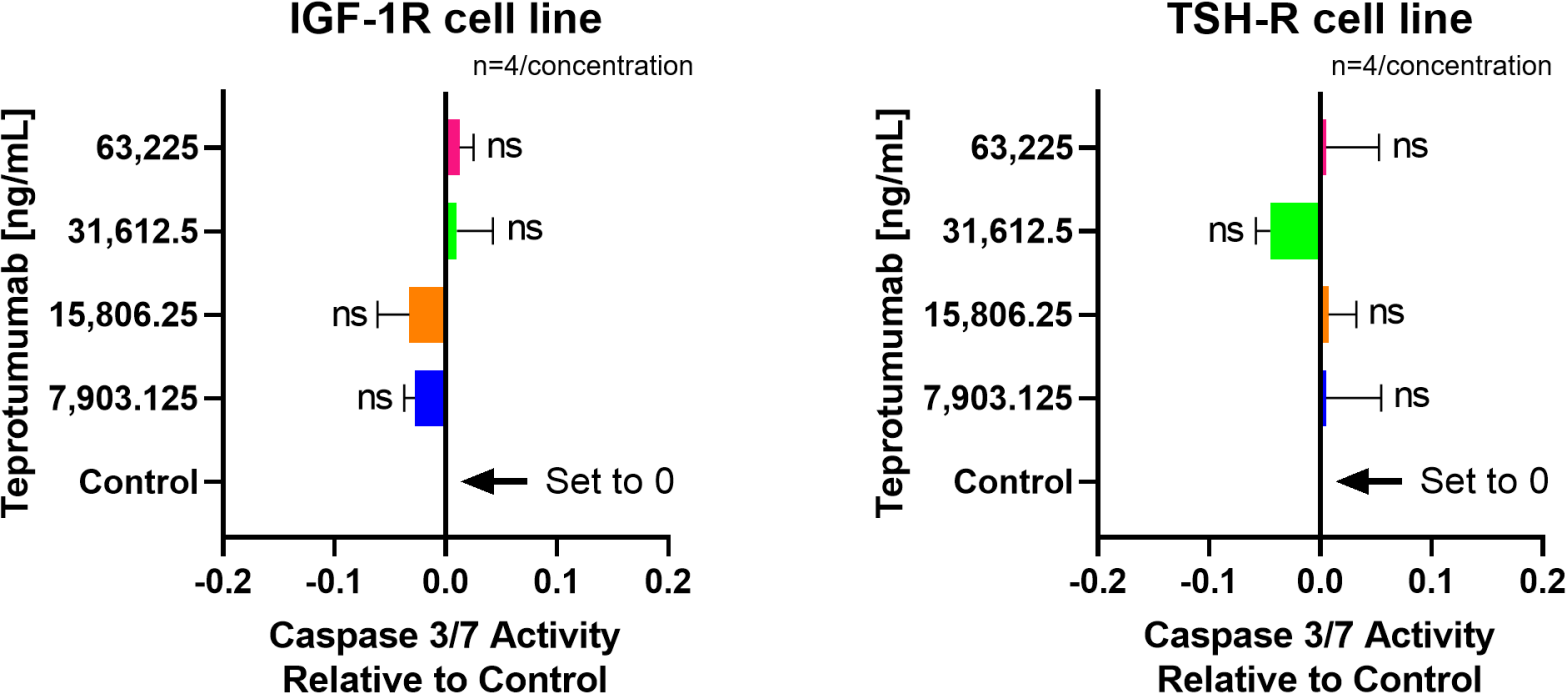
No effect of Teprotumumab on Caspase-3/7 Activity across all tested concentrations in IGF-1R and TSH-R cell lines. Caspase-3/7 activity ranged from -0.03 to +0.01 relative to control in the IGF-1R cell line and from -0.05 to +0.01 relative to control in the TSH-R cell line. Each concentration of Teprotumumab was tested in quadruplicate (n=4). ns means P > 0.05.

Linsitinib significantly increased caspase-3/7 activity in both IGF-1R and TSH-R cell lines. The caspase-3/7 activity ranged from +2.75 to +6.68 relative to control in the IGF-1R cell line and from +2.75 to +5.75 relative to control in the TSH-R cell line (**Figure 6**). Specifically, Linsitinib markedly increased caspase-3/7 activity at concentrations of 31,612.5 ng/mL (IGF-1R cell line +6.24 relative to control, P=0.0158, TSH-R cell line +5.53 relative to control, P=0.0048) and 63,225 ng/mL (IGF-1R cell line +6.68 relative to control, P=0.0005, TSH-R cell line +5.75 relative to control, P=0.0020). At lower concentrations (7,903.125 ng/mL and 15,806.25 ng/mL), no significant increase in caspase-3/7 activity was observed in either cell line. In contrast, Teprotumumab did not significantly increase caspase-3/7 activity across all tested concentrations in both cell lines. Caspase-3/7 activity ranged from -0.03 to +0.01 relative to control in the IGF-1R cell line and from -0.05 to +0.01 relative to control in the TSH-R cell line (**Figure 7**).

### 3.3 TSH-R-Ab bioassays

**Figure 8:**
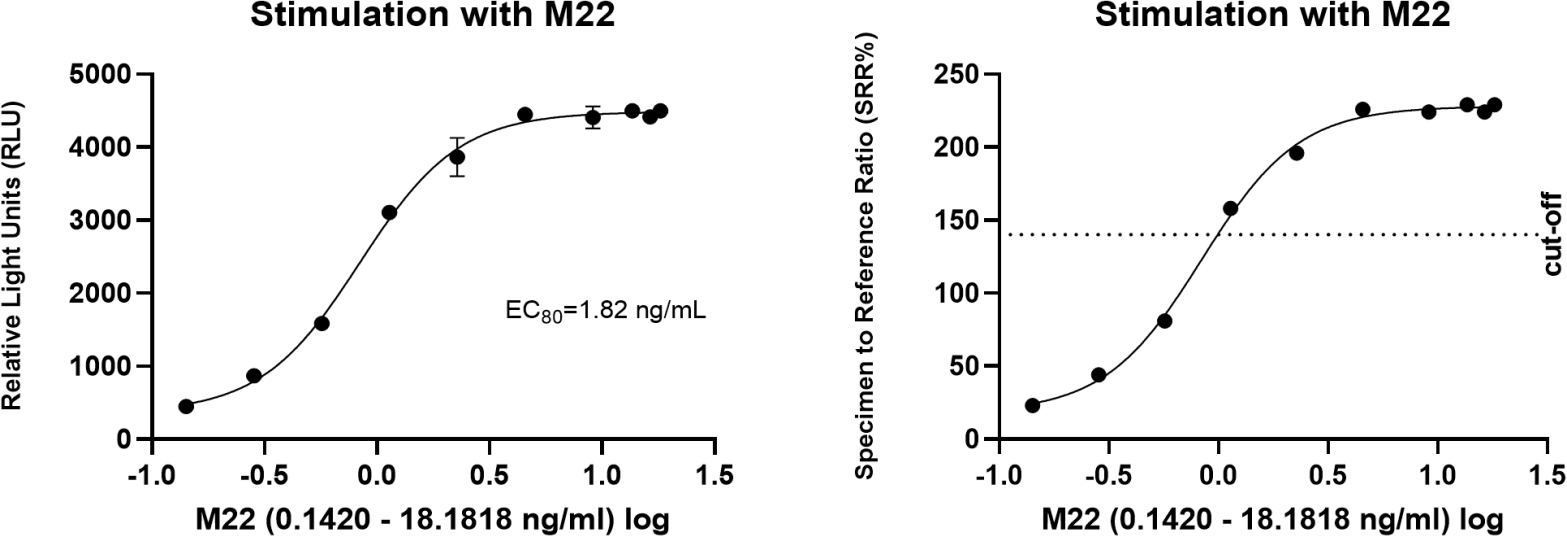
Dose-response curves for M22 in cell-based Thyretain TSH-R-Ab Stimulating (TSI) bioassay. An EC_80_ of 1.82 ng/mL of M22 was observed. Ten (n=10) rising concentrations of M22 were tested. The recorded relative light units (RLU) were converted to percentage of specimen-to-reference ratio (SRR %) with a cut-off of ≥ 140 SRR percentage.

**Figure 9:**
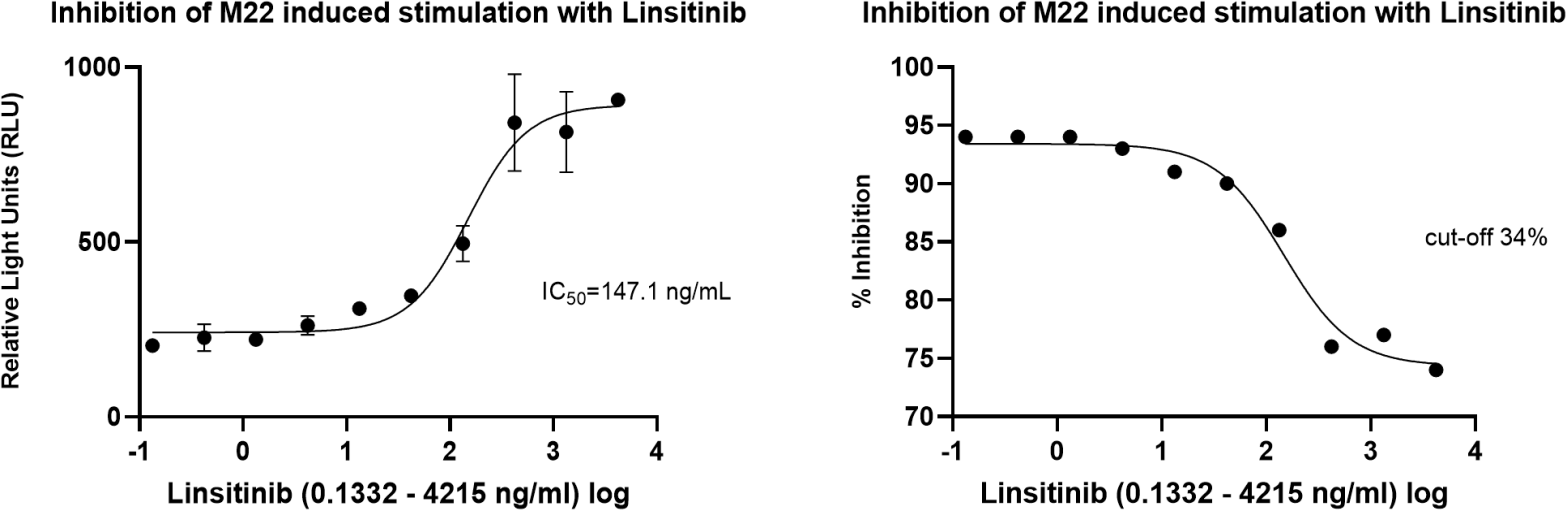
Dose-response curves for Linsitinib in cell-based Thyretain TSH-R-Ab Blocking (TBI) bioassay. An IC_50_ of 147.1 ng/mL of Linsitinib was observed. Ten (n=10) rising concentrations of Linsitinib were tested. The recorded relative light units (RLU) were reported as percent inhibition with a cut-off of ≥ 34%.

**Figure 10:**
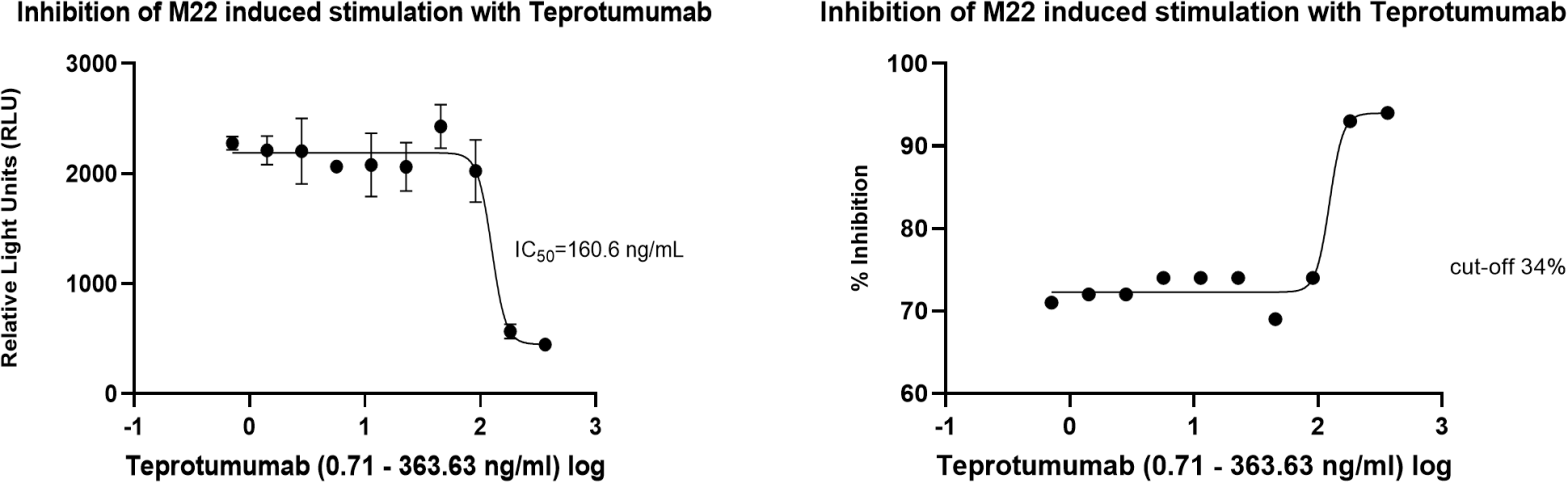
Dose-response curves for Teprotumumab in the cell-based TSH-R-Ab Blocking (TBI) bioassay. Teprotumumab showed an IC_50_ of 160.6 ng/mL. Ten (n=10) rising concentrations of Teprotumumab were tested. The recorded relative light units (RLU) were reported as percent inhibition with a cut-off of ≥ 34%.

In the cell-based TSI bioassay testing M22, seven out of 10 (70%) samples are positive, with their SRR percentage ranging from 158 to 238% (**Figure 8**). An EC_80_ of 1.82 ng/mL of M22 was observed. In the TBI blocking bioassay, all tested compounds demonstrated strong inhibition (100%) across all ten dilutions after induced stimulation by the previously determined double EC_80_ concentration of M22 (3.64 ng/mL). Linsitinib inhibition ranged from 74 to 94%, showing potent effects already at low concentrations (**Figure 9**), with an IC_50_ of 147.1 ng/mL. Notably, five out of 10 (50%) samples demonstrated inhibition ≥ 90%. Addition of Metformin to Linsitinib did not lower the IC_50_. Teprotumumab showed an IC_50_ of 160.6 ng/mL, with inhibition ranging from 69 to 94% (**Figure 10**). Highest positivity was registered at high concentrations of 181.81 ng/mL (93%) and 363.63 ng/mL (94%), respectively. As anticipated, the TSH-R blocking mAb K1-70 had the lowest IC_50_ of 20.57 ng/mL and demonstrated strong inhibition (98%) at high concentrations. In addition, four out of 10 (40%) samples showed inhibition ≥ 90% (**Supplementary Figure 5**).

## 4 Discussion

This pre-clinical study demonstrated for the first time, the potent inhibitory effect of the small molecule IGF-1R inhibitor Linsitinib on both IGF-1R and TSH-R expressing cell lines. Both qualitative morphological relevant changes of the cultured cells were registered as well as a quantitatively marked and highly significant inhibition of cell proliferation. In addition, Linsitinib strongly induced apoptosis of both IGF-1R and TSH-R expressing cells. Furthermore, potent inhibition of M22-induced stimulation with Linsitinib was observed in a cell-based blocking TBI bioassay, with an IC_50_ comparable to that of Teprotumumab. Βeta arrestin 1 may also mediate the dual inhibitory effect of Linsitinib on both cell lines, which scaffolds the reported crosstalk between the two major receptors in TED. Subsequent to binding of the small molecule Linsitinib to the ß subunit (ATP-binding domain) of the IGF-1 tyrosine kinase receptor, ß-arrestin 1 is recruited. Beta arrestin promotes binding of the E3 ubiquitin ligase Mouse double minute 2 homolog (Mdm2) to the IGF-1R. Subsequently, ubiquitination induces proteosomal degradation of the IGF-1R. Beta arrestin 1 also modulates the ERK signaling pathway. In comparison, monoclonal antibodies e.g. Teprotumumab particularly inhibit the PI3K/AKT signaling pathway. Overall, the pivotal role of ß-arrestin in mediating the reported crosstalk between both key receptors requires further investigation to assess its contribution to the dual inhibitory effect of Linsitinib.

In other words, these findings suggest that Linsitinib inhibits not only IGF-1R and TSH-R but also the crosstalk between these two key players. In line with our data, Linsitinib strongly inhibited TSH-R/IGF-1R crosstalk in TED fibroblasts, evidenced by the suppression of M22 mAb-stimulated hyaluronic acid secretion (27), hence highlighting its potential as a promising treatment for TED.

Linsitinib is a potent dual ATP-competitive kinase inhibitor of IGF-1R as well as IR (16, 19) and binds to the intracellular ATP-binding site of the kinase domain in an ATP-competitive fashion (18). This inhibition prevents the auto phosphorylation of the activation loop in the kinase domain, thereby blocking ligand-dependent activation of IGF-1R by IGF-1 and IGF-2 (16). Linsitinib has demonstrated antitumor activity, showing therapeutically efficacy across multiple pre-clinical tumor models (18, 28). Specifically, OSI-906-treated colorectal cancer xenografts exhibited decreased tumor growth and increased apoptosis in both *in vivo* and *in vitro* studies (29). Linsitinib’s inhibitory effects on cell growth were also highlighted in the human H295R cell line, which overexpresses the IGF-1R, and in the genetically identical HAC15 cell line, alone and in combination with mammalian target of rapamycin (mTOR) inhibitors (28). In addition, Linsitinib inhibited the proliferation of human adrenocortical carcinoma cell lines *in vitro* at lower concentrations than *in vivo* in humans (28). In hepatocellular carcinoma cell lines, OSI-906 reduced proliferation by at least 40% (30). Of note, Linsitinib has already reached clinical investigation and was tested in a phase III clinical trial (NCT00924989) for both patients with locally advanced adrenocortical carcinoma (31) as well as with advanced cell lung cancer (32).

Pertaining to its potential role in autoimmune endocrine and/or thyroidal disease, Linsitinib effectively prevented autoimmune hyperthyroidism in its early stages in an animal model and demonstrated a dose-dependent reduction in orbital adipogenesis (15). Furthermore, Linsitinib blocked bone marrow activation, inhibiting progression of TED, by reducing proinflammatory cytokines and inflammation in a mouse model (33).

Compared to Linsitinib, anti-IGF-1R monoclonal antibodies, such as Teprotumumab, target the cysteine-rich region of the IGF-1R extracellular domain with high-affinity and specificity (34). By binding to this extracellular region, Teprotumumab effectively blocks the binding pocket of the receptor, thereby preventing ligand-induced activation of downstream signaling pathways including AKT and ERK (16, 34). As previously mentioned, the anti-IGF-1R small molecules e.g. Linsitinib and monoclonal antibodies i.e. Teprotumumab use different blocking mechanisms: Linsitinib targets the intracellular while Teprotumumab targets the extracellular domain of the IGF-1R. Further experiments are of course warranted and foreseen to explore and confirm the novel and challenging findings of a markedly stronger inhibition of cell proliferation and an exclusive Linsitinib-induced apoptosis of both IGF-1R and TSH-R expressing cells. Noteworthy, the relevant function of β-arrestin 1, which can be modulated by other small molecules (35), should also be looked at.

Several current limitations of this pre-clinical study should be discussed. The experiments were conducted on cell lines, only. A larger sample size is being tested to confirm the robustness and reproducibility of the presented results. In addition, experiments with cultured primary human cells, e.g. orbital target cells (fibroblasts, pre-adipocytes, etc.) are ongoing to enhance the relevance of the results. Furthermore, ongoing experiments are assessing the toxicity of Linsitinib and Teprotumumab in both IGF-1R and TSH-R-expressing cells as well as in human orbital fibroblasts.

In the cell-based blocking TSHR-Ab bioassay, combined treatment with Linsitinib + Metformin was included to evaluate potential synergistic effects. Metformin is known to modulate IGF-1R downstream signaling pathways also targeted by Linsitinib. In this bioassay, we used a cell line overexpressing TSH-R, with much lower expression of IGF-1R, which may explain the lack of Metformin’s effect in this study. Additionally, the response to Metformin is highly dependent on glucose conditions. Metformin shows stronger inhibitory effects under low or physiological glucose levels. Future experiments will test Linsitinib + Metformin combination in low-glucose medium to evaluate Metformin’s additive effects.

In conclusion, Linsitinib’s potent induction of apoptosis and inhibition of proliferation in IGF-1R and TSH-R expressing cells highlights its promising therapeutic potential for effectively managing TED.

## Data Availability

The original contributions presented in the study are included in the article/**Supplementary Material**. Further inquiries can be directed to the corresponding author.

## Author’s note

The cell lines present in this study were obtained from [ATCC, American Type Culture Collection, Manassas, VA, USA] for the IGF-1R cell line, and from [QuidelOrtho, San Diego, CA, USA] for the TSH-R cell line.

## Supplementary material

Supplementary Figure 1 Procedure of the cell proliferation assay.

Supplementary Figure 2 Pipetting scheme for cell proliferation and caspase-Glo^®^ 3/7 Assay.

Supplementary Figure 3 Pipetting scheme for M22 testing in cell-based TSH-R-Ab Stimulating (TSI) bioassay.

Supplementary Figure 4 Pipetting scheme for testing IGF-1R and TSH-R inhibitors in cell-based TSH-R-Ab Blocking (TBI) bioassay.

Supplementary Figure 5 Dose-response curves for K1-70 in cell-based Thyretain TSH-R-Ab Blocking (TBI) bioassay.
