# Supplementary material for: Linsitinib inhibits proliferation and induces apoptosis of both IGF-1R and TSH-R expressing cells
Source: Front Immunol. 2024 Dec 11;15:1488220. doi: 10.3389/fimmu.2024.1488220 (PMC11668815; doi:10.3389/fimmu.2024.1488220)
Supplement: Supplementary Figure 1 — Procedure of the cell proliferation assay. [file Presentation1.pptx]

## Slide 1
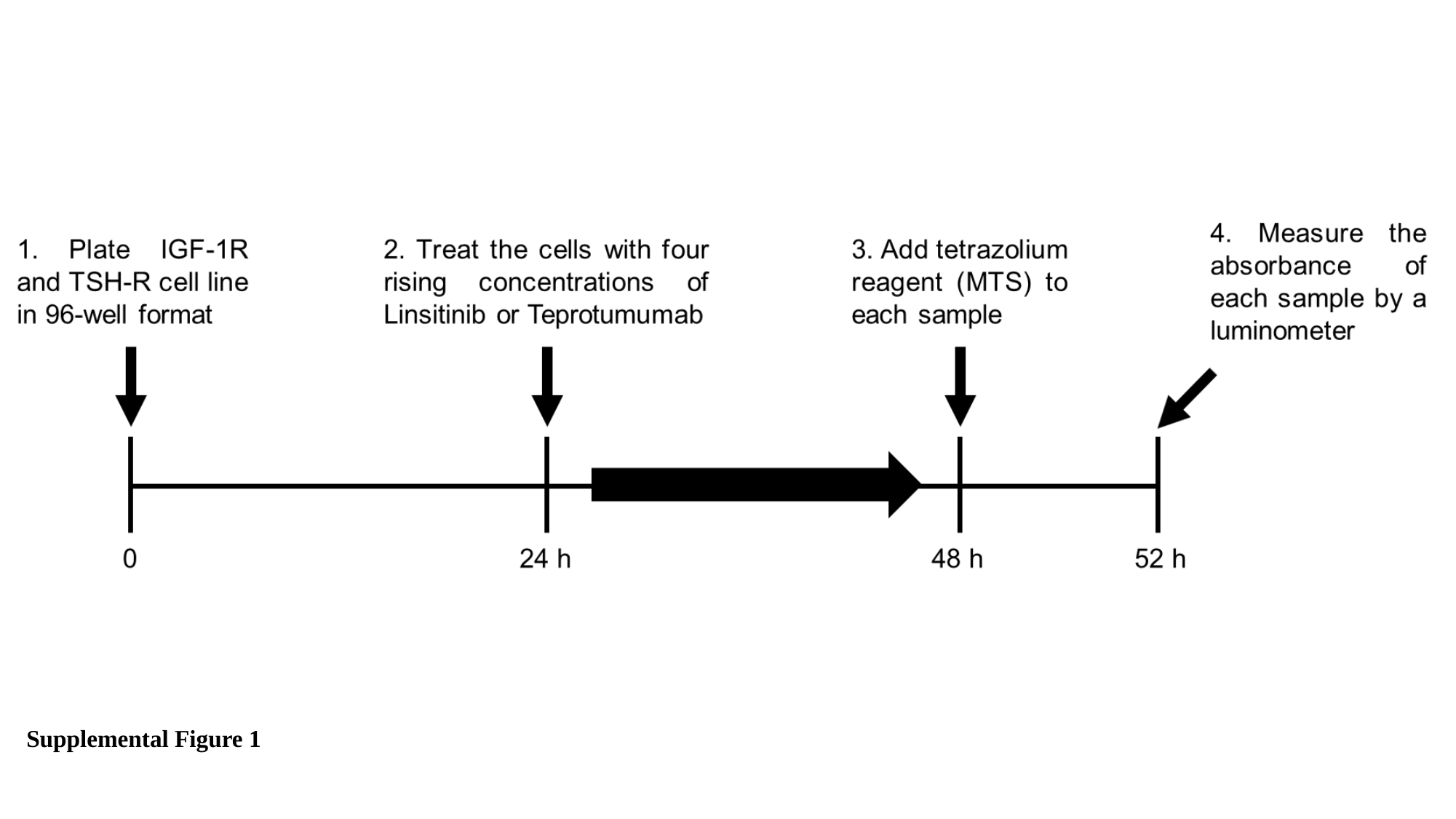

Supplemental Figure 1

## Slide 2
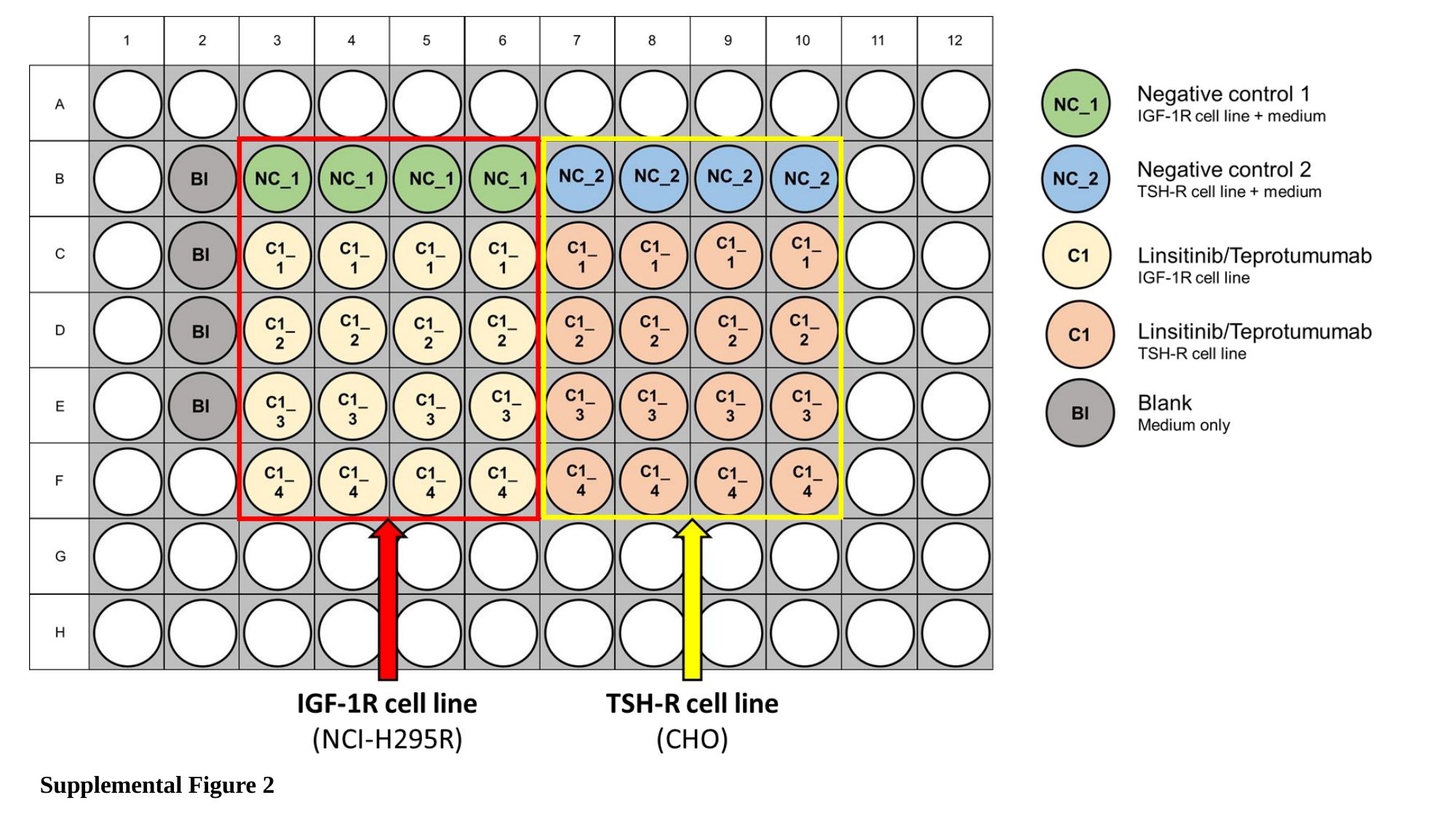

Supplemental Figure 2

## Slide 3
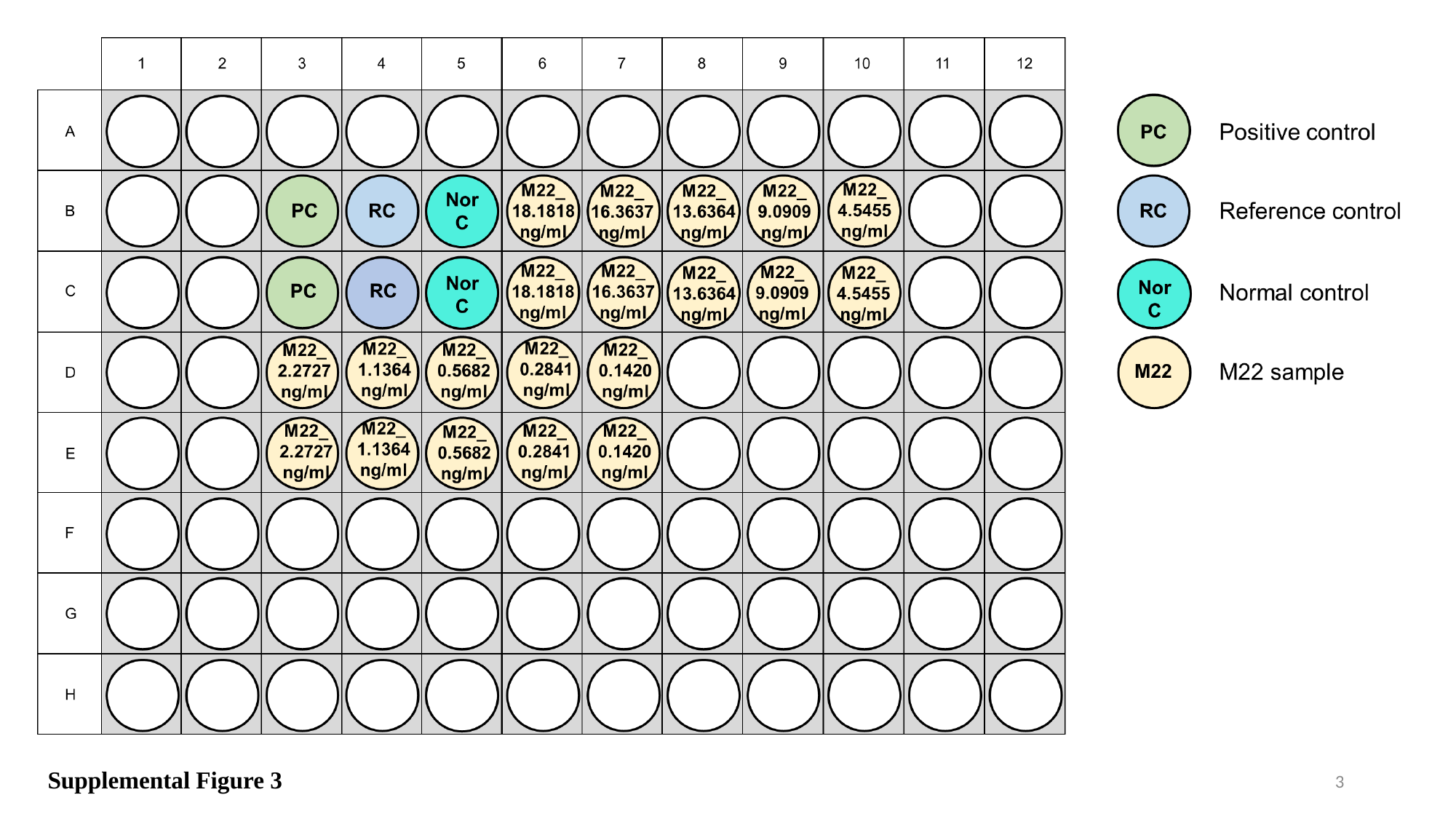

Supplemental Figure 3
3

## Slide 4
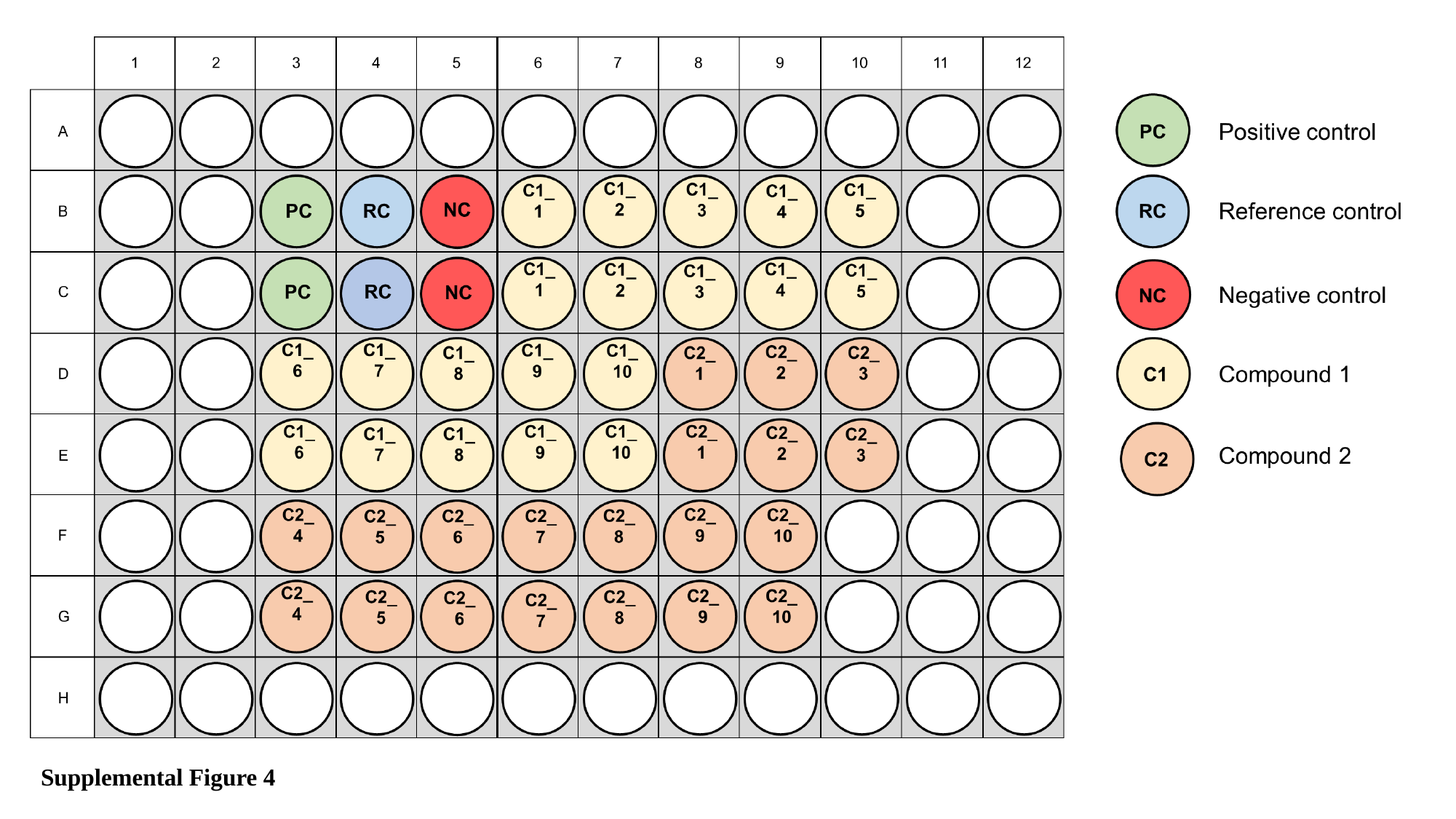

Supplemental Figure 4

## Slide 5
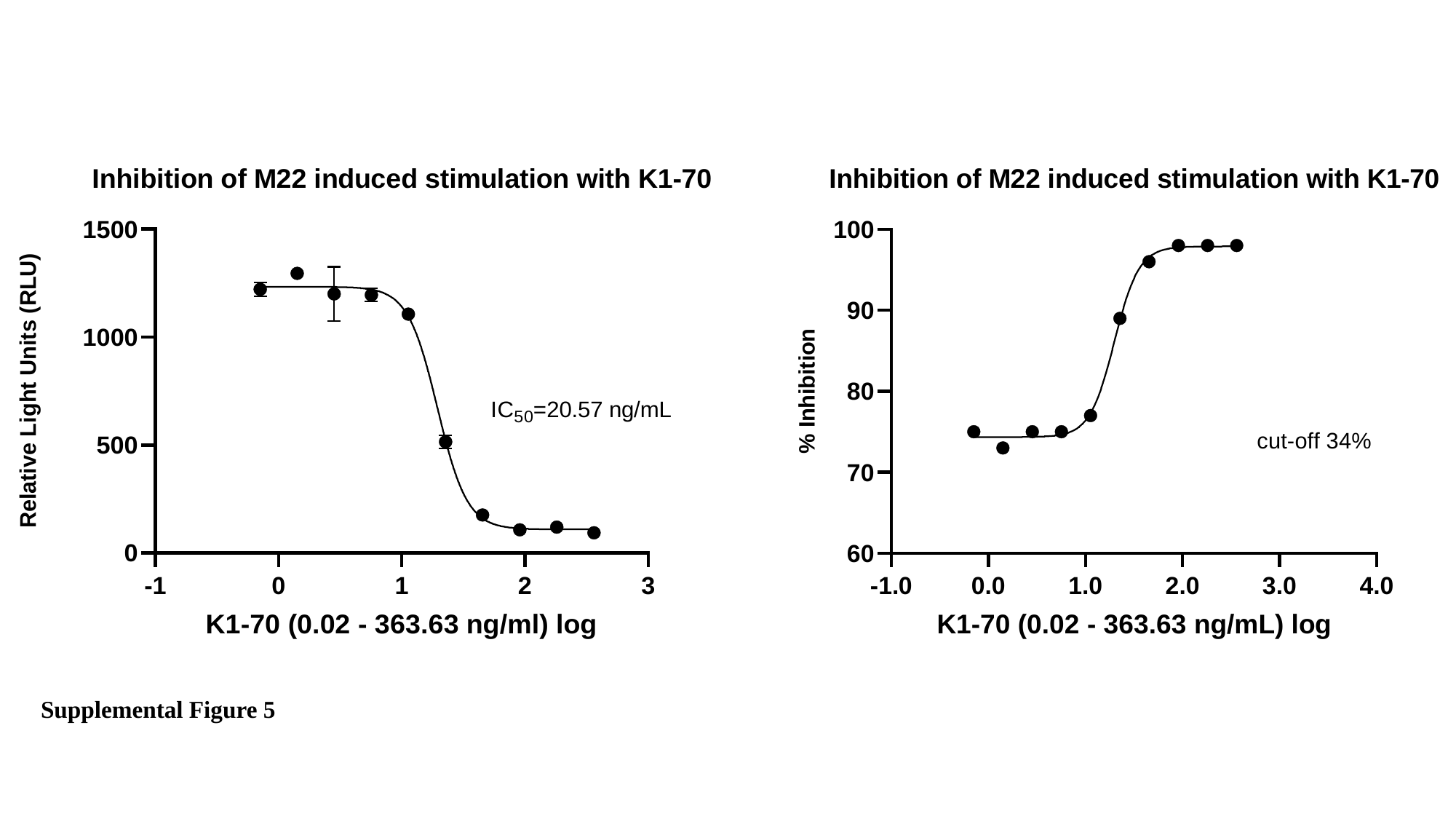

Supplemental Figure 5
